# Medical Education—New Professorships in the Medical Department of Harvard University

**Published:** 1866-07

**Authors:** 


### MEDICAL EDUCATION—NEW PROFESSORSHIPS IN THE MEDICAL DEPARTMENT OF HARVARD UNIVERSITY.

That medical education in America is rapidly changing its character may be seen by a glance at the long prospectuses sent out from the principal schools of our chief Atlantic cities. Until within a few years the whole public instruction of the student was limited to an annual four months' course of lectures, and the number of instructors everywhere corresponded to the six or eight general departments into which medicine had been divided for centuries. During the rest of the year he was left to manage his own education as he chose, and at the end of three years, a period of probation which was only nominally required by certain faculties, if he could pass a ridiculously easy examination in a certain proportion of these departments, he got his degree of Doctor of Medicine. With this necessarily general groundwork of knowledge, and without any proper clinical or special training, he went out into the world, either to make himself by observation, reading and natural shrewdness the really excellent, practical family physician, such as nearly every village in America may boast of, or to remain through life a very ignorant and cheap doctor. The few who fortunately had the means went at once after graduation to famous European schools, where medicine seemed a strange, almost new science to them, and where years might be spent in learning what was there taught in but a single subdivision of one of the old general departments.

Probably none were more sensible of, or more regretted the defects in our system of medical education, than those who have been professionally engaged in it here in New England, and efforts were made by them under the only general authority recognized, our national Association, to raise its character by a mutual agreement on the part of the schools in the country to insist upon a full three years' course of study and a more thorough examination before conferring degrees, but such a change threatened the financial prosperity of some of them by interfering with the chief source of their popularity, and nothing was effected. It was evident that without a central governing power all action in this direction must be individual. Harvard University had already taken the first important step by extending its course of instruction so as to cover the whole year in the medical, as in all its other great departments of study, and this system has been quite generally imitated within a few years by other large schools. It is, however, still non-obligatory, and although the summer sessions, so-called, are rapidly becoming more popular as their great importance is more widely appreciated, the custom remains everywhere in operation of conferring a degree upon any person who has had his name entered with any sort of a physician for three years and has bought tickets for eight months of lectures, provided he can pass a very simple examination in a certain number of the branches taught. In a country which boasts of its system of popular education as the best in the world, and where the interval which should always separate professional from general knowledge, if respect for the former is to be preserved, must necessarily be maintained by corresponding extraordinary progress in the former, in that country alone is medical education limited to the short term of three years. It has been found impossible, as has been said, to lengthen it by general agreement between the schools, but it should at least be made incumbent on their part that the most should be made of this brief period of training by using the whole year for instruction, and on the part of the student, that he should be obliged to attend the summer as well as the winter course. So much, we think, the American Medical Association may demand, and we doubt if any school now would dare object to a step which would not interfere with their own success and which would contribute so materially to elevate professional rank amongst us.

It should also insist upon students passing a satisfactory examination in every department before he can obtain a degree. If these branches, each and all, are considered as an essential part of a medical education, of course no person is fitted for practice until he has acquired the slight knowledge of them necessary to pass the examination at any of our schools. Full diplomas of Medicine Doctor are given, however, in cases where the candidate is so ignorant in the elementary branches of knowledge as to fail to pass in several of them.

Another and a far more important change in our system of medical education is the increase in the number of instructors in the schools within the last few years, It has become an impossibility for any man to know or to teach as a professor should, all that modern science has done in departments of medicine formerly included under a single head. In Europe it has long been the custom to divide and subdivide these branches, and instruction is there given not only by the numerous occupants of the regular chairs, but by so-called extraordinary professors and special instructors. Our own University has been among the first to recognize the necessity of labor, and has accordingly created the system of adjunct professors, assistant professors, and assistants, so that more than twenty instructors are now engaged in teaching in this department, in which several were considered sufficient, less than ten years ago.

